# A U.S. based survey of loan burden among anesthesia trainees and its impact on well-being

**DOI:** 10.3389/feduc.2025.1480957

**Published:** 2025-07-23

**Authors:** Niti Pawar, Christy K. Boscardin, David Chen, Gillian Earnest, Odmara L. Barreto Chang

**Affiliations:** 1 Department of Anesthesia and Perioperative Care, University of California San Francisco, San Francisco, CA, United States; 2 Department of Medicine, University of California San Francisco, San Francisco, CA, United States

**Keywords:** loan burden, underrepresented in medicine (URiM), first-generation college (FGC), female, anesthesia, residents, trainees, well-being

## Abstract

**Introduction:**

Loan burden presents a significant barrier for trainees in different medical fields. However, disparities in loan burden of anesthesia trainees of underrepresented in medicine (URiM), first-generation and female-identifying backgrounds have not been studied. Moreover, it is not known whether membership in these groups is associated with well-being or life decisions after controlling for loan burden.

**Methods:**

In this cross-sectional observational study, an online survey was disseminated by the American Society of Anesthesiologists (ASA) to anesthesia trainees in the United States (U.S.) from October to November 2022. Demographic information, Harvard Mental Health Continuum-Short Form (MHC-SF) well-being scores, and whether trainees perceive loan burden as delaying buying a house or having children were all collected. We evaluated associations between demographic group memberships, probability of having high loan burden, and delayed life decisions and well-being before and after adjusting for high loan burden.

**Results:**

The study represents 952 of 6,502 U.S. trainees (14.6%). The respondents had a mean age of 31 years, and 385 identified as female (40.4%), 150 as URiM (15.8%), and 634 as first-generation college (FGC) trainees (66.6%). The proportion of trainees with high loan burden was higher in the URiM 138 (92.0%) and FGC groups 565 (89.1%). Trainees in the high loan burden group were more likely to delay having children (OR = 3.69, 95% Simultaneous Confidence Interval (SCI) 2.38–5.73) and delay buying a home (OR = 5.27, 95% SCI: 3.45–8.05). Once loan burden was adjusted for, many disparities persisted in associations: URiM delaying buying a home (OR = 2.14, 95% SCI: 1.01–4.54), FGC delaying buying a home (OR = 1.56, 95% SCI: 1.02–2.38), female-identifying delaying children (OR = 1.49, 95% SCI: 1.04–2.13) and female-identifying well-being (Diff = −3.47, 95% SCI: −5.83, −1.11).

**Conclusions:**

FGC and URiM anesthesia trainees have significantly higher odds of having high loan burden and continue to experience disparities in life decisions after controlling for loan burden. Female-identifying trainees have significantly higher odds of delaying childbearing and have lower well-being after controlling for loan burden.

## 1 Introduction

Over the last decades, loan burden after medical school has increased dramatically in the United States (U.S.) (Greysen et al., 2011). In 2009, Collier et al. reported that the median loan burden in anesthesia residents upon medical school graduation was $113,746 (Collier et al., 2009). As of 2023, the median loan burden among medical school graduates is $215,100—double in comparison (Hanson, 2023).

The impact of loan burden has been studied in medical students and residents of across specialties attributing to negative impacts on life decisions, careers, and well-being. Pisaniello et al. found that higher debt in medical students was associated with poorer academic performance, an increased likelihood to pursue higher-paying specialties and a greater prevalence of alcohol dependence (Pisaniello et al., 2019). Among surgical residents, debt was perceived as a significant financial burden both during and after residency, influencing job choice, location of residence and practice, delaying buying a home, getting married, having children, and correlated with feeling less financially secure currently and about their future (*p* < 0.05) (Gray et al., 2020). A study on the impact of loan burden on well-being in anesthesia residents found that higher amounts of student debt were statistically significantly associated with a higher risk of distress and depression, with a 1% higher risk for each additional $10,000 owed (odds ratio = 1.01) (Sun et al., 2019). While this study addressed burnout, depression, and distress in anesthesia residents, it did not explore how the severity of loan burden influences these outcomes, or whether those first generation to college (FGC) or underrepresented in medicine (URiM) trainees are differentially impacted. Another key study with anesthesia residents found that higher debt was significantly associated with greater desire to moonlight (*p* = 0.002), decreased interest in academic careers (*p* < 0.001), and stronger preference for a residency with an education debt repayment program (*p* < 0.001) (Steiner et al., 2012). However, this study did not stratify any findings by FGC or URiM groups or assess the impact of loan burden on well-being.

Yet, several studies outside the field of anesthesia have evaluated disparities in loan burden, well-being, and career among FGC and URiM groups. Factors leading to increased loan burden in racial and ethnic minority groups included lower parental education, diminished financial resources, income, and wealth (Addo et al., 2016; Dugger et al., 2013). McMichael et al. found that URiM medical students had significantly higher odds of debt and the factors associated with higher levels of debt included race, first-generation status, and parental education (McMichael et al., 2022). Moreover, they reported that financial stress was significantly higher in females, URiM students, and FGC students (McMichael et al., 2022). Similarly, among female physicians in physical medicine and rehabilitation (PM&R), Black or African American doctors had significantly higher debt (p=0.005), and those in the highest quartile of debt had greater burnout (p=0.024), although no significant differences in race/ethnicity were found in comparison to the lower quartiles (Verduzco-Gutierrez et al., 2021). In a separate study, Baker and Barker reported that physicians whose parents had lower education and income were more likely to report that debt negatively impacted them (p < 0.05), even upon controlling for debt levels (Baker and Barker, 1997). Additionally, another study found that Black medical students were more likely to delay having children due to debt (Adjusted OR 0.47, 95% CI: 0.24, 0.94), were more likely to report that educational debt caused a high level of stress (Adjusted OR 0.39, 95% CI: 0.20, 0.76), and females were twice as likely to express concern about paying back loans (Rohlfing et al., 2014). A survey on dermatology applicants revealed that URiM students had significantly higher median debt ($200,000) compared to White students ($180,000, p<0.01), along with significantly lower United States Medical Licensing Examination (USMLE) Step 1 and 2 scores, fewer publications, and a lower match rate (URiM 76.6% vs. White 88.4%, p = 0.03) contributing to persistent underrepresentation in the specialty (Costello et al., 2022).

Ultimately, while prior studies have documented the negative impacts of loan burden, as well as disparities in loan burden among URiM and FGC groups and their impacts on well-being, there remains a lack of research that integrates all these variables within the field of anesthesiology. As a high-stress field with a high rate of burnout (59.2% of responding anesthesiologists were at high risk of burnout and 13.8% met criteria for burnout syndrome) (Afonso et al., 2021), resident well-being is crucial not only for individual health but also for patient safety. Notably, a study found that anesthesia residents at high risk for burnout and depression had a significantly higher incidence of medication errors compared to those at low risk (33% vs. 0.7%) (De Oliveira et al., 2013), which can have detrimental results in the operating room. As burnout, depression and suicide ideation is highly prevalent in anesthesiology residents, there is great need to address trainee well-being (De Oliveira et al., 2013). This may render anesthesiology residents especially vulnerable to additional stressors such as debt and financial pressure, that can further impact well-being and impact patient care.

Previously, Steiner et al. illustrated the detrimental impacts of high loan burden on anesthesia residents’ careers (Steiner et al., 2012), however a difference in loan burden or well-being by race and ethnicity or first-generation status has not yet been studied in anesthesia (Jette et al., 2021; Sadhasivam et al., 2012; Thomas et al., 2024; Willer et al., 2023). We hypothesized that URiM and First Generation College (FGC) anesthesia trainees would have higher loan burden, and that those with high loan burden would have impacted life decisions and lower well-being. In this study, we also evaluated whether URiM, FGC and female anesthesia trainees would continue to have disparities in life decisions and well-being after controlling for loan burden.

## 2 Materials and methods

### 2.1 Participants and design

This cross-sectional survey study was approved by the Institutional Review Board of the University of California, San Francisco (IRB 21–34959). The research was conducted in accordance with the Checklist for Reporting Results of Internet E-Surveys (CHERRIES) guidelines. The survey was developed by adapting questions from prior survey-based studies in anesthesia and other fields (Gray et al., 2020; Rohlfing et al., 2014; Steiner et al., 2012). Following the initial development of the survey, pilot testing was conducted among four anesthesia residents to gather feedback and ensure clarity in the wording of the questions.

In collaboration with the American Society of Anesthesiologists (ASA), a secure, web-based survey was administered to 6,502 anesthesia trainees through their email list. The survey was sent out by the ASA twice, 1 week apart, during October and November 2022. To enhance the response rate, the research team reached out to the 159 allopathic anesthesia residency programs recognized by the ASA in the U.S. and Puerto Rico, inviting them to disseminate the survey directly to their residents and fellows in training. Participation in the 4-min Qualtrics survey was voluntary, and the participants’ identities remained anonymous to the research team. Survey responses were securely stored on the UCSF server. As an incentive, participants who completed the survey were entered into a drawing for gift cards. The participants’ email addresses were retained solely as a means of contact for the drawing. Survey responses were carefully screened and duplicates based on email and IP address were removed.

### 2.2 Survey content

The survey was administered over the Qualtrics online survey software program. The survey items included demographic information, loan burden, and well-being. Demographic information included age, year of training (Clinical Anesthesia (CA)1-CA 4, representing the years of anesthesia residency before fellowship), race and ethnicity, and gender. Options for indicating gender were “Cisgender female/woman,” “Cisgender male/man,” “Transgender female/woman,” “Transgender male/man,” and “Gender not listed here,” with a free text entry. For race and ethnicity, trainees self-identified in several categories: “African American or Black,” “Asian,” “Hispanic or Latino/x,” “American Indian or Alaska Native,” “Native Hawaiian or Pacific Islander,” “White or Caucasian,” and “Other (Free text entry).” Respondents were able to pick multiple categories. The definition of underrepresented in medicine (URiM) was based on the American Association of Medical Colleges (AAMC): “Racial and ethnic populations that are underrepresented in the medical profession relative to their numbers in the general population” that previously referred to “Black, Mexican-American, Native American (American Indian, Alaska Native, and Native Hawaiian), and mainland Puerto Rican populations” (Association of American Medical Colleges). All other ethnicities were classified as non-URiM. Furthermore, FGC trainees were defined as the first in their families to complete a college or university degree and were analyzed as a dichotomous variable.

To evaluate loan burden, we provided the following brackets for participants to select: “Less than 50,000,” “50,000–100,000,” “100,001–200,000,” “200,001–300,000,” and “More than 300,000.” For analysis as a dichotomous variable, high loan burden was categorized as >$100,000, and low loan burden as ≤$100,000. This cutoff was selected based on the findings by Phillips et al., which indicated that physicians graduating from public schools were more likely to practice primary care and family medicine if their loan burden was <$100,000 (p<0.01) (Phillips et al., 2014). The relationship persisted even when socioeconomic status was examined separately, indicating a difference in mindset at the $100,000 mark (Phillips et al., 2014).

To measure well-being, we utilized the Harvard Mental Health Continuum-Short Form (MHC-SF), a validated and widely recognized survey instrument. This tool consists of 70 total points, divided into three sub-scores: hedonic (emotional well-being, 0–15 points), eudaimonic (social, 0–25 points), and psychological (well-being, 0–30 points) (Lamers et al., 2011). The 14 items are scored on a Likert scale from 0 to 5 to calculate subscale scores and an overall total score. Higher scores reflect greater levels of well-being. This framework ideologizes that well-being encompasses both the absence of negative emotions and the presence of positive emotions, measuring how frequently participants experience each.

Additional questions regarding the perceived impact of loan burden on life decisions and future planning were asked, similar to those in the previous survey conducted by Gray et al. (2020). Respondents provided answers using a Likert scale of five options: “strongly disagree,” “disagree,” “neutral,” “agree,” and “strongly agree,” which were scored from 1 to 5, respectively. For analysis, responses were categorized as dichotomous with “Strongly Disagree to Neutral” (1–3) = 0 and “Agree to Strongly Agree” (4–5) = 1. For example, “Repaying debt. . . delays buying a house, having children” was assessed with a Likert scale where “Strongly Disagree to Neutral” (1–3) = 0 (No/Low) and “Agree to Strongly Agree” (4–5) = 1 (Yes/High). Questions were not randomized or alternated, nor was there any adaptive questioning. There were about 4–6 questions per page over 4 pages.

### 2.3 Statistical analysis

**FIGURE 1:**
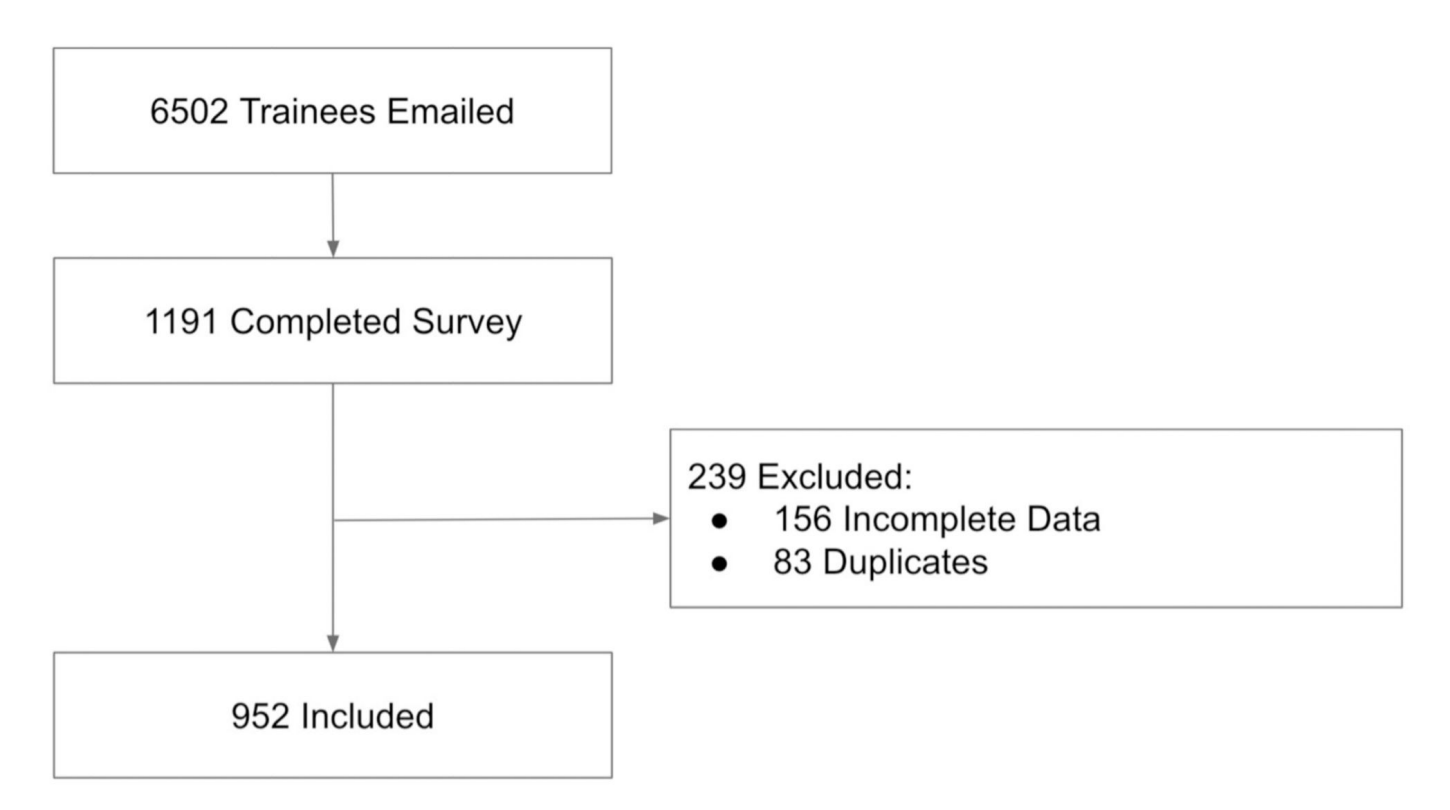
Recruitment flowchart.

Participants with incomplete (*n* = 156) or duplicate entries based on IP address and email (*n* = 83) were excluded from the analysis (Figure 1). Incomplete responses were characterized as those missing information on loan burden, race and ethnicity, first-generation status, gender, and/or well-being scores. Due to an insufficient number of responses from nonbinary individuals (*N* = 7, 0.6%), the gender comparison in the analyses was kept to cisgender women and cisgender men. Among the excluded responses, the missing variables included loan burden (*n* = 134), race and ethnicity (*n* = 78), first-generation status (*n* = 69), gender (*n* = 73), MHC-SF well-being score (*n* = 134), and age (*n* = 72).

To evaluate the associations between the predictors of being URiM, FGC, and female-identifying with the probability of having high loan burden, we employed univariate logistic regressions to compute a set of odds ratios, with 95% simultaneous confidence intervals (c = 2.40) calculated using non-parametric bootstrap methods (Montiel Olea and Plagborg-Møller, 2019). Similarly, to investigate the association between high loan burden and the outcomes of well-being, delaying having children, and buying a home, we used univariate logistic regressions to compute a set of odds ratios, with 95% simultaneous confidence intervals computed by non-parametric bootstrap (c = 2.39). To obtain loan burden-adjusted associations between the predictors (URiM, FGC, and female-identifying) and the outcomes (delaying buying a home and having children, and MHC well-being score), we used a set of 9 regressions adjusted for each predictor, loan burden, and their respective 2-way interactions. Logistic regressions were employed to estimate the expected probabilities of delaying home buying and having children, with associations quantified as odds ratios. Linear regressions were used to estimate expected well-being scores, with associations quantified by score differences. We computed 95% simultaneous confidence intervals for the 9 estimates (c=2.80), again using nonparametric bootstrap. All analyses were performed using R Statistical Software (v4.1.2; R Core Team 2021). To control for the type I family-wise error rate of 0.05 across multiple comparisons, we utilized a 95% simultaneous confidence interval (SCI). Intervals that do not include null effects (1 for odds ratios or 0 for differences) were considered statistically significant.

## 3 Results

Of the 6,502 total trainees in the United States who received the survey through the ASA or their residency program, there were 1,191 responses (18.3% response rate), of which 952 had complete data (14.6%). The mean age of respondents was 31 years, 385 (40.4%) were female-identifying, 150 were URiM (15.8%), and 634 were FGC (66.6%, Table 1). The demographics of the survey sample by race and ethnicity as well as gender are comparable to the national demographics of anesthesia residents based on data from the Accreditation Council for Graduate Medical Education (ACGME) 2023–24 data (Accreditation Council for Graduate Medical Education).

### 3.1 Associations between demographic groups and loan burden

Of the URiM trainees, there were more trainees with a high loan burden than a low loan burden (138, 92.0% vs. 12, 8.0%). This also extended to the subgroups of Black (69, 98.6% vs. 1, 1.4%), Hispanic (57, 85.1% vs. 10, 14.9%), American Indian or Alaska Native (9, 100.0% vs. 0, 0.0%), and Native Hawaiian or Other Pacific Islander trainees (3, 75.0% vs. 1, 25.0%). Relatively, the proportions of trainees with high loan burden were less for the Asian (180, 75.6% vs. 58, 24.4%) and White groups (456, 80.9% vs. 108, 19.1%) when comparing to the URiM group. Also, more FGC trainees had a high loan burden (565, 89.1%) rather than a low loan burden (69, 10.9%). URiM (OR = 3.00, 95% SCI: 1.36–6.64) and FGC trainees (OR = 4.27, 95% SCI: 2.80–6.52), were significantly more likely to have higher loan burden at a family-wise error rate (FWER) of 0.05.

The odds ratio for female-identifying trainees compared to male-identifying trainees was not statistically significant at the 0.05 FWER (OR=0.87, 95% SCI: 0.58–1.30, Table 2).

### 3.2 Impact of loan burden on life decisions and well-being

**FIGURE 2:**
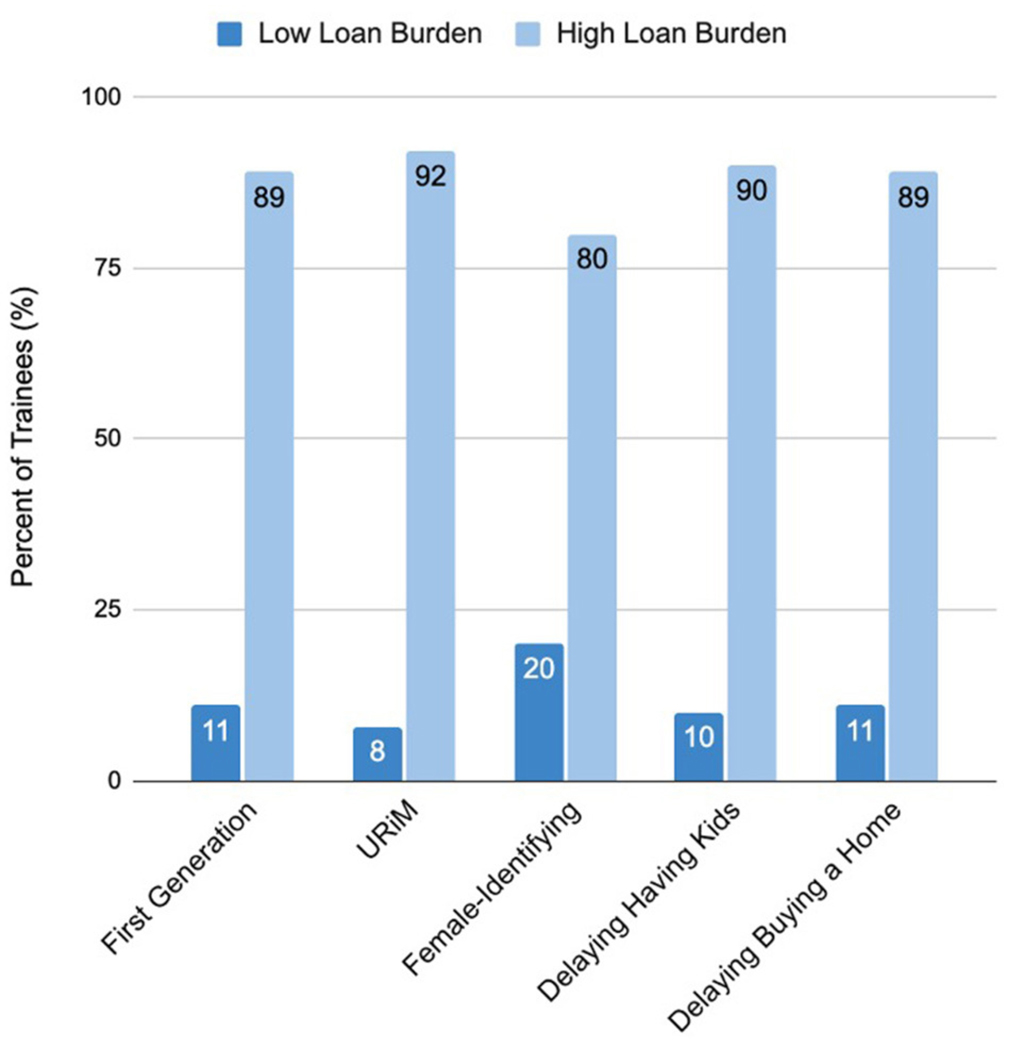
Percentage of trainees in different categories (low vs. high loan burden).

Trainees in the high loan burden group were more likely to report delaying having children (457, 90.1%) and delaying buying a home (611, 89.2%). Figure 2 illustrates these differences in outcomes according to loan burden. For well-being, the average total MHC score of the high loan burden group was 44 compared to 46 in the low loan burden group (Table 1).

Those with high loan burden were more likely at a FWER of 0.05 to delay having children (OR = 3.69, 95% SCI 2.38–5.73) and buying a home (OR = 5.27, 95% SCI: 3.45–8.05, Table 3). Having high vs. low loan burden was associated with an estimated 1.54 point drop in expected well-being score. However, this difference was not statistically significant at the FWER of 0.05 (Diff = −1.54, 95% SCI: −3.89–0.80, Table 3).

### 3.3 Associations between demographic group memberships and life decisions and well-being after controlling for loan burden

After adjusting for loan burden and loan burden interactions with the groups of interest (URiM, FGC, and female-identifying trainees), there were persistent, statistically significant disparities at an FWER of 0.05: delaying buying a home in URiM trainees (OR = 2.14, 95% SCI: 1.01–4.54), delaying buying a home in FGC trainees (OR = 1.56, 95% SCI: 1.02–2.38), delaying children in female-identifying trainees (OR = 1.49, 95% SCI: 1.04–2.13) and lower well-being in female-identifying trainees (Diff = −3.47, 95% SCI: −5.83, −1.11, Table 4).

## 4 Discussion

In this study, we evaluated the impact of loan burden on anesthesia trainees’ well-being, career choices, and lifestyle decisions to better understand the barriers contributing to inequities within the field. While previous research has examined loan burden, particularly its effect on FGC and URiM groups, no study has yet explored the interactions among these variables within the context of anesthesia trainees. This work contributes to the expanding literature on the impact of unequal access to higher education among medical trainees and further investigates how disparities persist, even after accounting for loan burden.

Our findings build on previous studies on loan burden by illustrating that URiM and FGC anesthesia trainees are more likely to experience higher loan burden. One potential inference from these studies is that increasing financial burden may deter individuals from marginalized backgrounds from pursuing medicine, thereby contributing to further underrepresentation in the field of medicine and anesthesia. Additionally, financial strain persists beyond the entry into medical practice, as evidenced by a study examining residents across various specialties, including surgery and family medicine. Holaday et al. (2023) found that ethnoracial disparities in educational debt exacerbate racial wealth gaps even among high-income earners; URiM residents are more likely to have accumulated debt prior to medical school and require longer periods to repay medical training debt (Holaday et al., 2023). Thompson and Gustavo (2019) noted that these discrepancies between income and wealth are particularly pronounced among URiM trainees (Thompson and Gustavo, 2019). Interestingly, there was a substantial increase in medical school applications following the COVID-19 pandemic, attributed to a rise in applicants qualifying for fee waivers and the elimination of travel expenses (Boyle, 2021). URiM applicants led this surge, as Black or African American applicants rose by 21.0% from 2020–21, followed by an 8.3% increase in Asian applicants and a 7.1% increase in Hispanic applicants (Boyle, 2021).

Furthermore, our findings indicate that FGC and URiM trainees experience significant disparities on their life decisions and overall well-being. Specifically, FGC and URiM trainees were more likely to delay buying homes independently of loan burden. Notably, FGC trainees also had lower well-being scores and were more likely to delay having children compared to their counterparts independently of loan burden, though these differences did not reach statistical significance. These results suggest that factors beyond financial constraints, such as psychological and social influences, contribute to these disparities. In one study, deficiencies in parental education, income, and expectations have been shown to reduce the likelihood of URiM children attaining a college education and associated with decreased interest and preparedness for medical school (Cooper, 2003). While the MHC-SF has not been used previously in anesthesia residents for comparison, a study in mixed-specialty first-year residents found an average score of 49.29 at the start of residency, compared to our averages of 46 and 44 in the low and high loan burden groups (Lebares et al., 2021). These results suggest that systemic factors may persist to adversely affect well-being mediated by increased financial strains and lead to a decrease in the number of URiM and FGC students entering anesthesia.

Addressing the loan burden in URiM and FGC trainees is crucial, as it can negatively impact the pipeline into medicine and exacerbate racial inequities and underrepresentation. Compared to the proportion of Black and Hispanic ethnicities in the United States general population in 2024 (33.2%) (United States Census Bureau), a study from 2023 highlights that Black and Hispanic anesthesia residents comprise only 9.6%, with representation further declining among anesthesia professors to 3.9% (Armaneous et al., 2023). URiM faculty members also continue to occupy lower academic ranks (Reece-Nguyen et al., 2023). Disparities within anesthesiology begin at the application stage. A study spanning 2011 to 2022 found that females had significantly lower odds of applying to anesthesia (*P* < 0.0001) despite similar odds of matching, while URiM applicants had significantly lower odds of matching despite similar odds of applying (*P* < 0.001) (Sumarli et al., 2024). Trainees also encounter disparities in board exam scores based on their racial and ethnic backgrounds. A study in 2025 found significant differences in anesthesiology BASIC first-time board exam scores, illustrating that the odds of passing were significantly lower for female-identifying individuals (OR = 0.53, 95% confidence interval [CI], 0.47–0.60), Black/African American individuals (OR = 0.41, 95% CI, 0.33–0.51), and Hispanic or Latino individuals (OR = 0.52, 95% CI, 0.42–0.64) (Sun et al., 2025). Despite multiple factors from K-12 through medical school contributing to the underrepresentation of URiM and female minorities in anesthesiology (Chiem et al., 2022; Odonkor et al., 2022), one persistent threat to their career advancement is the burden of student loans.

Lastly, our study highlighted the disparities faced by female-identifying anesthesia trainees. Female-identifying trainees had significantly lower well-being and were significantly more likely to delay having children independently of loan burden. Similarly, other studies in medicine have reported that female physicians experience more symptoms of burnout compared to male physicians (De Oliveira et al., 2013; Gisselbaek et al., 2023, 2024), a phenomenon associated with career choice regret (Dyrbye et al., 2018). A recent publication addressing the difficulties faced by URiM and female physician-scientists advocated for an increase in student loan forgiveness as one strategy to improve diversity (Ward et al., 2022).

Regarding the postponement of childbearing, it is important to recognize the biological differences in reproductive capacity between males and females; while males do not face a restricted window of fertility, females have a limited timeframe for childbearing (Dunson et al., 2002). These findings are crucial, as delayed childbearing may be one contributing factor to decreased well-being. A recent study found that compared to the general population, female physicians were significantly older at first pregnancy, more likely to have infertility, miscarriages, and preterm births, and reported discouragement from “negative workplace attitudes regarding pregnancy” (Lai et al., 2023). Additionally, a study of surgical residents found that female residents more often experienced pregnancy and parenthood-related mistreatment as well as obstetric complications, both of which were associated with higher burnout and thoughts of attrition (Li et al., 2024). The challenges associated with pregnancy during anesthesia training may exacerbate the disparities faced by female anesthesiologists. Disparities are evident in female representation in the workforce, senior academic ranks, authorship, grant awards, speaking at national meetings, editorial board membership, department, and national society leadership, along with a lower lifetime earning potential (Emala et al., 2023; Esslinger et al., 2020; Gisselbaek et al., 2023; Reece-Nguyen et al., 2023; Toledo et al., 2020; Weeks et al., 2007).

These results, along with the underrepresentation of women in the anesthesia workforce, academia, and leadership (Esslinger et al., 2020; Toledo et al., 2020) suggest that childbirth may be a significant factor contributing to this disparity. The financial component of childbirth is equally important to note. A study surveying female-identifying members of the American Society of Anesthesiologists (ASA) found that income loss related to maternity leave, negatively affected 19.5% of pregnant residents’ abilities to financially support their family (Kraus et al., 2021). Historically, childbearing has posed significant challenges for women in medicine, highlighting an area requiring further improvement. Programs should consider ways to counter the negative culture around pregnancy in medicine to support trainees during their reproductive years and improve trainee well-being and equity in the field. Adequate paid parental leave was among the top three desired changes for physician mothers (Adesoye et al., 2017). Additional interventions include offering benefits for fertility preservation (University of California Family-Forming Program), financial support for pregnancy-related leaves of absence (Lai et al., 2023), adopting competency-based medical education, building in coverage of trainees to decrease the burden of leave on colleagues, and providing lactation accommodations for physicians in practice (Buyske and Hawn, 2022).

### 4.1 Limitations

This study has several limitations and cannot establish causality; instead, it describes associations between various outcomes and membership in groups of interest. A larger sample size may have improved our ability to detect more statistically significant effects where we currently observe trends (i.e., lower well-being and delaying children in FGC). The unequal distribution of low vs. high loan burden (178 vs. 774) may expose odds ratio estimates to the risk of sparse data bias. However, our sensitivity analyses indicated that the significant associations reported largely persisted across high vs. low loan burden cutoffs, resulting in more balanced inferences. Another limitation is the using a dichotomous classification rather than a quartile-based or continuous classification, which was chosen for statistical simplicity. Additionally, it is not possible to ascertain whether the reported perceptions of future outcomes will translate into actual outcomes. Nevertheless, these perceptions during the residency are crucial to understanding and comparing URiM and non-URiM trainees, as they influence career trajectories, i.e., the likelihood of pursuing a career in academia. To increase diversity with the number of female and URiM trainees in academia, research, and leadership positions, residency represents a pivotal intervention stage for supporting their career-decision making process.

Lastly, future studies should explore how URiM, FGC, and female-identifying groups impact job choice when adjusted for loan burden (i.e., more likely to enter private practice, primary care, or academia). A deeper understanding of the interplay between loan burden and factors related to URiM, FGC, and female-identifying identities would advance progress toward a more equitable and representative workforce and inform the development of targeted interventions. For example, restructuring loan financing could mitigate the psychological impact of loan burden and support URiM and FGC trainees with higher debt (Garrett et al., 2022; Young et al., 2016). Additionally, providing financial education may enhance anesthesia trainees’ financial literacy, planning skills, and ability to manage loan burdens more effectively.

One notable strength of this study is its demonstration of disparities in well-being and life decisions among URiM, FGC, and female-identifying trainees, even after controlling for a high loan burden. Although the rate of response was 18.3%, it is comparable to prior surveys conducted by the American Society of Anesthesiologists among U.S. residents (Afonso et al., 2021; Hertzberg et al., 2021; Vandenberg et al., 2023). The study’s findings have significant implications and underscore the importance of recognizing the critical differences among trainee groups. In our analysis, we accounted for loan burden to evaluate the impact of demographic variables. This approach helped to mitigate the potential bias of convenience sampling, under the assumption that individuals with higher loan burdens were more likely to complete the survey. We believe that the selection process does not undermine the generalizability of our findings. Therefore, we believe the study’s external validity extends to anesthesia trainees across the United States.

## 5 Conclusion

Among anesthesia trainees in the United States, those from FGC and URiM backgrounds bear significantly higher loan burdens compared to their counterparts. This higher level of debt affects overall well-being and influences their life decisions. Notably, well-being and life decisions are negatively impacted regardless of loan burden in trainees who identify as FGC, URiM, or female, illustrating further disparities. These results underscore the importance of addressing the disparities in loan burden and other factors impacting URiM, FGC, and female-identifying trainees to improve the representation and diversity within anesthesia.

## Figures and Tables

**TABLE 1:** Demographics characteristics of anesthesia trainees (*N* = 952).

Variables	Low loan burden *N* = 178	High loan burden *N* = 774	*p*-value
Age in years^a^	31 (4.9)	31 (3.3)	0.667
Female-identifying	77 (20.0)	308 (80.0)	0.401
**Race and ethnicity** ^a^			
Asian	58 (24.4)	180 (75.6)	0.016
Black	1 (1.4)	69 (98.6)	<0.001
Hispanic	10 (14.9)	57 (85.1)	0.376
Native American or Pacific Islander	1 (7.7)	12 (92.3)	0.168
White	108 (19.1)	456 (80.9)	0.666
URiM	12 (8.0)	138 (92.0)	<0.001
**Training stage** ^a^			
Intern	31 (23.8)	99 (76.2)	0.136
CA1	52 (22.2)	182 (77.8)	0.129
CA2	35 (15.8)	187 (84.2)	0.182
CA3	28 (15.6)	152 (84.4)	0.207
≥ CA4	32 (17.2)	154 (82.8)	0.552
FGC	69 (10.9)	565 (89.1)	<0.001
Delaying having children	50 (9.9)	457 (90.1)	<0.001
Delaying buying home	74 (10.8)	611 (89.2)	<0.001
Harvard MHC-SF^a^	46 (11.6)	44 (12.9)	0.118

Data are *n* (%) or

a Mean (standard deviation).

*p*-value is by *t*-test calculation.

MHC-SF, mental health continuum short form; CA1, clinical anesthesia year 1; URiM, underrepresented in medicine, FGC, first generation college.

**TABLE 2:** Differences in loan burden by demographic groups.

High loan burden	
Variable	OR (95% SCI)
URiM	3.00 (1.36, 6.64)^∗^
FGC	4.27 (2.80, 6.52)^∗^
Female-identifying	0.87 (0.58, 1.30)

Data are OR, odds ratio (95% SCI, simultaneous confidence interval).

∗ *p* < 0.05.

URiM, underrepresented in medicine, FGC, first generation college.

**TABLE 3:** Differences in life decisions and well-being by loan burden.

	Delay having children	Delay buying home	Well-being
Variable	OR (95% SCI)	OR (95% SCI)	Diff (95% SCI)
High loan burden	3.69 (2.38, 5.73)*	5.27 (3.45, 8.05)*	−1.54 (−3.89, 0.80)

Data are OR, odds ratio (95%, SCI, simultaneous confidence interval) and difference in expected well-being MHC-SF scores (95% simultaneous confidence interval, SCI).

* *p* < 0.05.

**TABLE 4:** Models of life decisions and well-being by demographic groups after adjustment for loan burden.

	Delaying having children	Delaying buying home	Well-being
	OR (95% SCI)	OR (95% SCI)	Diff (95% SCI)
URiM	1.01 (0.59, 1.73)	2.14 (1.01, 4.54)*	−0.24 (−3.09, 2.61)
FGC	1.37 (0.92, 2.03)	1.56 (1.02, 2.38)*	−1.35 (−3.85, 1.15)
Female-identifying	1.49 (1.04, 2.13)*	1.04 (0.71, 1.53)	−3.47 (−5.83, −1.11)*

Data are OR = odds ratio (95% SCI, simultaneous confidence interval) and difference in expected well-being MHC-SF scores (95% SCI, simultaneous confidence interval).

* *p* < 0.05.

URiM, underrepresented in medicine, FGC, first generation college.

## Data Availability

The raw data supporting the conclusions of this article will be made available by the authors, without undue reservation.

## Appendix I.

Mental Health Continuum – Short Form (MHC – SF) (Lamers et al., 2011) In the past month, how often did you feel. . .

1. Happy
2. Interested in life
3. Satisfied
4. That you had something important to contribute to society
5. That you belonged to a community (like a social group, your neighborhood, your city)
6. That our society is becoming a better place for people
7. That people are basically good
8. That the way our society worked makes sense to you
9. That you liked most parts of your personality
10. Good at managing the responsibilities of your daily life
11. That you had warm and trusting relationships with others
12. That you have experiences that challenge you to grow and become a better person
13. Confident to think or express your own ideas and opinions
14. That your life has a sense of direction or meaning to it

Responses evaluated with Likert Scale from Strongly Disagree to Strongly Agree

## Abbreviations:

URiM: underrepresented in medicine
FGC: first generation college
UCSF: University of California, San Francisco
ASA: American Society of Anesthesiologists
AAMC: American Association of Medical Colleges
MHC-SF: Mental Health Continuum Short Form
IRB: Institutional Review Board
CHERRIES: checklist for reporting results of internet E-surveys
SCI: simultaneous confidence interval
OR: odds ratio
CA1-CA4: clinical anesthesia year 1 to 4
FWER: family-wise error rate
COVID-19: coronavirus disease 2019
ACGME: accreditation council for graduate medical education
U.S.: United States
USMLE: United States medical licensing examination
